# Bis(4,6-dimethyl­pyrimidine-2-thiol­ato)dimethyl­tin(IV)

**DOI:** 10.1107/S1600536810030412

**Published:** 2010-08-28

**Authors:** Yang Shi, Ru-Fen Zhang, Chun-Lin Ma

**Affiliations:** a College of Chemistry and Chemical Engineering, Liaocheng University, Shandong 252059, People’s Republic of China

## Abstract

The asymmetric unit of the title complex, [Sn(CH_3_)_2_(C_6_H_7_N_2_S)_2_], contains two independent mol­ecules with similar configurations. In each, the Sn^IV^ cation is coordinated by two methyl and two 4,6-dimethyl­pyrimidine-2-thiol­ate anions in a distorted SnS_2_C_2_ tetra­hedral geometry. In the two mol­ecules, the S—Sn—S bond angles are 87.70 (5) and 88.93 (4)°, while the C—Sn—C bond angles are 125.7 (3) and 125.9 (2)°. Weak C—H⋯N hydrogen bonding is present in the crystal structure.

## Related literature

For applications of organotin compounds, see: Duboy & Roy (2003 ▶); Gielen (2002 ▶).
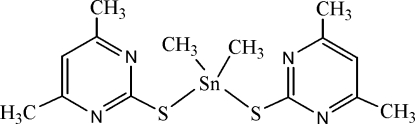


## Experimental

### 

#### Crystal data

[Sn(CH_3_)_2_(C_6_H_7_N_2_S)_2_]
*M* *_r_* = 427.15
Monoclinic, 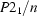

*a* = 10.5787 (17) Å
*b* = 26.731 (4) Å
*c* = 13.393 (2) Å
β = 91.001 (2)°
*V* = 3786.7 (11) Å^3^
*Z* = 8
Mo *K*α radiation
μ = 1.57 mm^−1^
*T* = 273 K
0.43 × 0.38 × 0.16 mm

#### Data collection

Bruker SMART CCD area-detector diffractometer
Absorption correction: multi-scan (*SADABS*; Sheldrick, 1996 ▶) *T*_min_ = 0.552, *T*_max_ = 0.787
19775 measured reflections
6678 independent reflections
4533 reflections with *I* > 2σ(*I*)
*R*_int_ = 0.037

#### Refinement

*R*[*F* ^2^ > 2σ(*F* ^2^)] = 0.036
*wR*(*F* ^2^) = 0.090
*S* = 1.06
6678 reflections
391 parameters
H-atom parameters constrained
Δρ_max_ = 0.40 e Å^−3^
Δρ_min_ = −0.50 e Å^−3^

### 

Data collection: *SMART* (Siemens, 1996 ▶); cell refinement: *SAINT* (Siemens, 1996 ▶); data reduction: *SAINT*; program(s) used to solve structure: *SHELXS97* (Sheldrick, 2008 ▶); program(s) used to refine structure: *SHELXL97* (Sheldrick, 2008 ▶); molecular graphics: *SHELXTL* (Sheldrick, 2008 ▶); software used to prepare material for publication: *SHELXTL*.

## Supplementary Material

Crystal structure: contains datablocks I, global. DOI: 10.1107/S1600536810030412/xu5005sup1.cif

Structure factors: contains datablocks I. DOI: 10.1107/S1600536810030412/xu5005Isup2.hkl

Additional supplementary materials:  crystallographic information; 3D view; checkCIF report

## Figures and Tables

**Table 1:** Hydrogen-bond geometry (Å, °)

*D*—H⋯*A*	*D*—H	H⋯*A*	*D*⋯*A*	*D*—H⋯*A*
C6—H6*A*⋯N6^i^	0.96	2.55	3.397 (7)	147

Symmetry code: (i) 
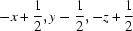
.

**Fig. 1.**
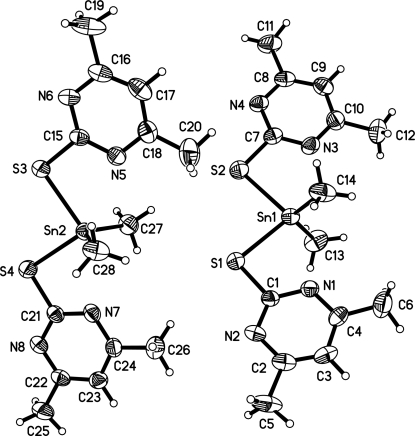
The asymmetric unit of the title compound, showing 30% probability displacement ellipsoids.

## supplementary crystallographic information

### Comment

In recent years, organotin complexes have attracted increasing attention owing to their wide industrial applications and biological activities (Duboy & Roy, 2003). In order to explore the relationships between these applications and their structures, a large number of organotin compounds have been prepared and studied (Gielen, 2002). In this connection, we report the structure of the title compound, (CH_3_)_2_Sn(SC_6_H_7_N_2_)_2_. As shown in Fig. 1, the title compound is a mononuclear dimethyltin(IV) derivate. The asymmetric unit contains two monomers. The structures of the two independent molecules are almost the same, with only small differences in bond lengths and bond angles. In the two molecules the S–Sn–S bond angles are 87.70 (5) and 88.93 (4)°, while the C–Sn–C bond angles are 125.7 (3) and 125.9 (2)°. Weak C—H···N hydrogen bonding is present in the crystal structure.

### Experimental

The reaction was carried out under nitrogen atmosphere. the 4,6-dimethyl-2-mercaptopyrimidine (0.280 g, 2 mmol), sodium ethoxide (0.136 g, 2 mmol) and dimethyltin dichloride (0.220 g, 1 mmol) was added in turn in benzene (20 ml), stirred for 12 h at 313 K and filtrated. The solvent was gradually removed by evaporation under vacuum until a solid product was obtained. Colorless crystals suitable for X-ray diffraction were obtained by recrystallization.

### Refinement

The H atoms were positioned geometrically, with methyl C—H distance of 0.96, aromatic C—H distance of 0.93 Å, and refined as riding on their parent atoms with *U*_iso_(H) = 1.2*U*_eq_(C,N) or 1.5*U*_eq_(C) for the methyl groups.

### Crystal data

**Table:**

[Sn(CH_3_)_2_(C_6_H_7_N_2_S)_2_]	*F*(000) = 1712
*M**_r_* = 427.15	*D*_x_ = 1.498 Mg m^−^^3^
Monoclinic, *P*2_1_/*n*	Mo *K*α radiation, λ = 0.71073 Å
Hall symbol: -P 2yn	Cell parameters from 6539 reflections
*a* = 10.5787 (17) Å	θ = 2.3–24.8°
*b* = 26.731 (4) Å	µ = 1.57 mm^−^^1^
*c* = 13.393 (2) Å	*T* = 273 K
β = 91.001 (2)°	Block, colorless
*V* = 3786.7 (11) Å^3^	0.43 × 0.38 × 0.16 mm
*Z* = 8	

### Data collection

**Table:**

Bruker SMART CCD area-detector diffractometer	6678 independent reflections
Radiation source: fine-focus sealed tube	4533 reflections with *I* > 2σ(*I*)
graphite	*R*_int_ = 0.037
φ and ω scans	θ_max_ = 25.0°, θ_min_ = 2.1°
Absorption correction: multi-scan (*SADABS*; Sheldrick, 1996)	*h* = −12→12
*T*_min_ = 0.552, *T*_max_ = 0.787	*k* = −31→31
19775 measured reflections	*l* = −14→15

### Refinement

**Table:**

Refinement on *F*^2^	Primary atom site location: structure-invariant direct methods
Least-squares matrix: full	Secondary atom site location: difference Fourier map
*R*[*F*^2^ > 2σ(*F*^2^)] = 0.036	Hydrogen site location: inferred from neighbouring sites
*wR*(*F*^2^) = 0.090	H-atom parameters constrained
*S* = 1.06	*w* = 1/[σ^2^(*F*_o_^2^) + (0.0349*P*)^2^ + 1.8858*P*] where *P* = (*F*_o_^2^ + 2*F*_c_^2^)/3
6678 reflections	(Δ/σ)_max_ = 0.001
391 parameters	Δρ_max_ = 0.40 e Å^−^^3^
0 restraints	Δρ_min_ = −0.50 e Å^−^^3^

### Special details

**Table:**

Geometry. All e.s.d.'s (except the e.s.d. in the dihedral angle between two l.s. planes) are estimated using the full covariance matrix. The cell e.s.d.'s are taken into account individually in the estimation of e.s.d.'s in distances, angles and torsion angles; correlations between e.s.d.'s in cell parameters are only used when they are defined by crystal symmetry. An approximate (isotropic) treatment of cell e.s.d.'s is used for estimating e.s.d.'s involving l.s. planes.
Refinement. Refinement of *F*^2^ against ALL reflections. The weighted *R*-factor *wR* and goodness of fit *S* are based on *F*^2^, conventional *R*-factors *R* are based on *F*, with *F* set to zero for negative *F*^2^. The threshold expression of *F*^2^ > σ(*F*^2^) is used only for calculating *R*-factors(gt) *etc*. and is not relevant to the choice of reflections for refinement. *R*-factors based on *F*^2^ are statistically about twice as large as those based on *F*, and *R*- factors based on ALL data will be even larger.

### Fractional atomic coordinates and isotropic or equivalent isotropic displacement parameters (Å^2^)

**Table:**

	*x*	*y*	*z*	*U*_iso_*/*U*_eq_	
Sn1	0.29835 (3)	0.070638 (12)	0.28079 (2)	0.05902 (12)	
Sn2	0.46690 (3)	0.321709 (12)	0.29062 (2)	0.05684 (11)	
N1	0.5146 (3)	0.04328 (14)	0.1909 (3)	0.0572 (10)	
N2	0.6789 (4)	0.10341 (16)	0.1758 (3)	0.0689 (11)	
N3	0.0515 (4)	0.05130 (16)	0.3491 (3)	0.0672 (11)	
N4	−0.0609 (4)	0.12016 (15)	0.4172 (3)	0.0648 (11)	
N5	0.2278 (4)	0.30583 (15)	0.2095 (3)	0.0604 (10)	
N6	0.0827 (4)	0.37434 (15)	0.2086 (3)	0.0645 (11)	
N7	0.7109 (4)	0.29191 (14)	0.3290 (3)	0.0594 (10)	
N8	0.8426 (4)	0.34338 (14)	0.4329 (3)	0.0588 (10)	
S1	0.47037 (13)	0.13220 (5)	0.26493 (12)	0.0772 (4)	
S2	0.17451 (14)	0.13676 (5)	0.36460 (12)	0.0814 (4)	
S3	0.30242 (12)	0.38749 (5)	0.30106 (11)	0.0700 (4)	
S4	0.61257 (13)	0.37658 (5)	0.38733 (11)	0.0730 (4)	
C1	0.5659 (4)	0.08862 (17)	0.2041 (3)	0.0568 (12)	
C2	0.7474 (5)	0.0685 (2)	0.1292 (4)	0.0752 (15)	
C3	0.7034 (5)	0.0213 (2)	0.1139 (4)	0.0775 (16)	
H3	0.7541	−0.0025	0.0838	0.093*	
C4	0.5828 (5)	0.00892 (18)	0.1432 (4)	0.0669 (14)	
C5	0.8762 (5)	0.0855 (3)	0.0965 (5)	0.117 (2)	
H5A	0.8669	0.1120	0.0486	0.175*	
H5B	0.9199	0.0579	0.0667	0.175*	
H5C	0.9239	0.0973	0.1534	0.175*	
C6	0.5228 (7)	−0.0405 (2)	0.1240 (5)	0.104 (2)	
H6A	0.4815	−0.0517	0.1833	0.156*	
H6B	0.5864	−0.0643	0.1059	0.156*	
H6C	0.4616	−0.0375	0.0705	0.156*	
C7	0.0413 (5)	0.09874 (19)	0.3781 (3)	0.0606 (12)	
C8	−0.1617 (5)	0.0906 (2)	0.4255 (3)	0.0651 (13)	
C9	−0.1586 (5)	0.0414 (2)	0.3959 (4)	0.0736 (15)	
H9	−0.2299	0.0213	0.4012	0.088*	
C10	−0.0492 (5)	0.0224 (2)	0.3586 (4)	0.0748 (15)	
C11	−0.2767 (5)	0.1137 (2)	0.4713 (4)	0.0980 (19)	
H11A	−0.2750	0.1493	0.4615	0.147*	
H11B	−0.2769	0.1065	0.5415	0.147*	
H11C	−0.3515	0.1001	0.4401	0.147*	
C12	−0.0373 (7)	−0.0313 (2)	0.3279 (6)	0.128 (3)	
H12A	−0.0235	−0.0332	0.2573	0.192*	
H12B	−0.1136	−0.0489	0.3436	0.192*	
H12C	0.0328	−0.0463	0.3631	0.192*	
C13	0.3509 (6)	0.0161 (2)	0.3880 (4)	0.0935 (19)	
H13A	0.3236	−0.0163	0.3655	0.140*	
H13B	0.3119	0.0237	0.4504	0.140*	
H13C	0.4412	0.0161	0.3969	0.140*	
C14	0.2190 (5)	0.0561 (3)	0.1390 (4)	0.0965 (19)	
H14A	0.2143	0.0206	0.1285	0.145*	
H14B	0.2708	0.0709	0.0888	0.145*	
H14C	0.1356	0.0702	0.1348	0.145*	
C15	0.1916 (4)	0.35227 (19)	0.2334 (3)	0.0570 (12)	
C16	0.0008 (5)	0.3462 (2)	0.1562 (4)	0.0708 (14)	
C17	0.0285 (6)	0.2986 (3)	0.1298 (4)	0.0819 (17)	
H17	−0.0302	0.2796	0.0938	0.098*	
C18	0.1440 (6)	0.2784 (2)	0.1566 (4)	0.0712 (14)	
C19	−0.1224 (5)	0.3714 (3)	0.1293 (5)	0.124 (3)	
H19A	−0.1638	0.3816	0.1892	0.187*	
H19B	−0.1756	0.3484	0.0929	0.187*	
H19C	−0.1064	0.4002	0.0887	0.187*	
C20	0.1851 (7)	0.2268 (2)	0.1290 (5)	0.112 (2)	
H20A	0.2350	0.2283	0.0699	0.168*	
H20B	0.1120	0.2063	0.1166	0.168*	
H20C	0.2347	0.2127	0.1827	0.168*	
C21	0.7356 (4)	0.33284 (17)	0.3837 (3)	0.0543 (11)	
C22	0.9334 (4)	0.30907 (19)	0.4280 (3)	0.0602 (12)	
C23	0.9162 (5)	0.26541 (18)	0.3745 (4)	0.0641 (13)	
H23	0.9798	0.2414	0.3728	0.077*	
C24	0.8040 (5)	0.25800 (17)	0.3239 (4)	0.0625 (13)	
C25	1.0534 (5)	0.3200 (2)	0.4843 (4)	0.0843 (16)	
H25A	1.0953	0.3479	0.4540	0.126*	
H25B	1.1075	0.2912	0.4828	0.126*	
H25C	1.0344	0.3281	0.5523	0.126*	
C26	0.7785 (6)	0.2129 (2)	0.2592 (5)	0.0972 (19)	
H26A	0.7000	0.1978	0.2779	0.146*	
H26B	0.8458	0.1892	0.2680	0.146*	
H26C	0.7733	0.2230	0.1905	0.146*	
C27	0.4156 (5)	0.26283 (19)	0.3870 (4)	0.0783 (15)	
H27A	0.3577	0.2408	0.3528	0.117*	
H27B	0.3760	0.2764	0.4450	0.117*	
H27C	0.4899	0.2445	0.4070	0.117*	
C28	0.5214 (5)	0.3140 (2)	0.1398 (3)	0.0823 (17)	
H28A	0.5344	0.2793	0.1249	0.123*	
H28B	0.5984	0.3322	0.1295	0.123*	
H28C	0.4560	0.3271	0.0966	0.123*	

### Atomic displacement parameters (Å^2^)

**Table:**

	*U*^11^	*U*^22^	*U*^33^	*U*^12^	*U*^13^	*U*^23^
Sn1	0.0535 (2)	0.0648 (2)	0.0589 (2)	0.00175 (16)	0.00344 (16)	0.00119 (16)
Sn2	0.0546 (2)	0.0580 (2)	0.0576 (2)	−0.00125 (16)	−0.00538 (15)	0.00102 (15)
N1	0.050 (2)	0.057 (2)	0.065 (3)	0.0019 (19)	0.0042 (19)	0.0066 (19)
N2	0.042 (2)	0.084 (3)	0.081 (3)	−0.006 (2)	0.004 (2)	0.002 (2)
N3	0.066 (3)	0.075 (3)	0.062 (3)	0.011 (2)	0.013 (2)	−0.014 (2)
N4	0.060 (3)	0.080 (3)	0.055 (2)	0.011 (2)	0.006 (2)	−0.010 (2)
N5	0.060 (3)	0.065 (3)	0.057 (2)	−0.003 (2)	−0.003 (2)	−0.001 (2)
N6	0.046 (2)	0.085 (3)	0.063 (3)	0.004 (2)	0.003 (2)	0.002 (2)
N7	0.059 (2)	0.056 (2)	0.062 (2)	−0.008 (2)	−0.011 (2)	0.004 (2)
N8	0.054 (2)	0.066 (2)	0.056 (2)	−0.011 (2)	−0.005 (2)	0.0046 (19)
S1	0.0581 (8)	0.0638 (8)	0.1101 (11)	0.0000 (6)	0.0133 (8)	−0.0147 (7)
S2	0.0663 (9)	0.0764 (9)	0.1021 (11)	0.0001 (7)	0.0200 (8)	−0.0143 (8)
S3	0.0536 (7)	0.0646 (8)	0.0914 (10)	−0.0002 (6)	−0.0108 (7)	−0.0110 (7)
S4	0.0604 (8)	0.0669 (8)	0.0910 (10)	0.0050 (6)	−0.0183 (7)	−0.0137 (7)
C1	0.046 (3)	0.059 (3)	0.065 (3)	0.003 (2)	−0.004 (2)	0.003 (2)
C2	0.052 (3)	0.106 (5)	0.068 (3)	0.003 (3)	0.000 (3)	0.004 (3)
C3	0.064 (4)	0.097 (4)	0.072 (4)	0.030 (3)	0.005 (3)	−0.004 (3)
C4	0.077 (4)	0.058 (3)	0.066 (3)	0.018 (3)	0.002 (3)	0.008 (2)
C5	0.048 (3)	0.178 (7)	0.125 (6)	−0.003 (4)	0.018 (4)	−0.005 (5)
C6	0.129 (6)	0.064 (4)	0.120 (5)	−0.002 (4)	0.020 (4)	−0.011 (3)
C7	0.060 (3)	0.074 (3)	0.047 (3)	0.012 (3)	0.005 (2)	−0.002 (2)
C8	0.053 (3)	0.097 (4)	0.046 (3)	0.012 (3)	0.006 (2)	−0.002 (3)
C9	0.064 (4)	0.092 (4)	0.066 (3)	−0.009 (3)	0.008 (3)	0.002 (3)
C10	0.074 (4)	0.078 (4)	0.073 (4)	−0.006 (3)	0.011 (3)	−0.011 (3)
C11	0.071 (4)	0.133 (5)	0.092 (4)	0.019 (4)	0.027 (3)	−0.012 (4)
C12	0.123 (6)	0.080 (5)	0.181 (7)	−0.007 (4)	0.012 (5)	−0.038 (5)
C13	0.107 (5)	0.094 (4)	0.080 (4)	0.019 (4)	0.008 (4)	0.032 (3)
C14	0.064 (4)	0.151 (6)	0.074 (4)	0.006 (4)	−0.004 (3)	−0.013 (4)
C15	0.047 (3)	0.070 (3)	0.054 (3)	−0.002 (2)	0.005 (2)	0.006 (2)
C16	0.051 (3)	0.108 (5)	0.054 (3)	−0.009 (3)	−0.003 (3)	0.007 (3)
C17	0.073 (4)	0.114 (5)	0.059 (3)	−0.027 (4)	−0.001 (3)	−0.008 (3)
C18	0.083 (4)	0.074 (4)	0.057 (3)	−0.011 (3)	0.005 (3)	−0.007 (3)
C19	0.057 (4)	0.207 (8)	0.108 (5)	0.012 (4)	−0.023 (4)	0.012 (5)
C20	0.150 (7)	0.088 (5)	0.098 (5)	−0.014 (4)	−0.010 (4)	−0.031 (4)
C21	0.052 (3)	0.060 (3)	0.051 (3)	−0.008 (2)	−0.008 (2)	0.006 (2)
C22	0.048 (3)	0.074 (3)	0.059 (3)	−0.009 (3)	0.003 (2)	0.013 (3)
C23	0.057 (3)	0.063 (3)	0.072 (3)	0.005 (2)	0.003 (3)	0.011 (3)
C24	0.066 (3)	0.052 (3)	0.070 (3)	−0.006 (3)	−0.002 (3)	0.000 (2)
C25	0.055 (3)	0.102 (4)	0.096 (4)	−0.008 (3)	−0.009 (3)	0.005 (3)
C26	0.096 (5)	0.069 (4)	0.126 (5)	0.001 (3)	−0.011 (4)	−0.020 (3)
C27	0.081 (4)	0.074 (4)	0.079 (4)	−0.004 (3)	−0.007 (3)	0.017 (3)
C28	0.067 (4)	0.125 (5)	0.055 (3)	0.000 (3)	0.006 (3)	−0.001 (3)

### Geometric parameters (Å, °)

**Table:**

Sn1—C14	2.099 (5)	C9—H9	0.9300
Sn1—C13	2.114 (5)	C10—C12	1.500 (7)
Sn1—S1	2.4654 (14)	C11—H11A	0.9600
Sn1—S2	2.4804 (14)	C11—H11B	0.9600
Sn2—C27	2.112 (5)	C11—H11C	0.9600
Sn2—C28	2.121 (5)	C12—H12A	0.9600
Sn2—S4	2.4766 (14)	C12—H12B	0.9600
Sn2—S3	2.4793 (13)	C12—H12C	0.9600
N1—C4	1.337 (6)	C13—H13A	0.9600
N1—C1	1.338 (5)	C13—H13B	0.9600
N2—C1	1.321 (5)	C13—H13C	0.9600
N2—C2	1.343 (6)	C14—H14A	0.9600
N3—C10	1.324 (6)	C14—H14B	0.9600
N3—C7	1.331 (6)	C14—H14C	0.9600
N4—C8	1.334 (6)	C16—C17	1.356 (8)
N4—C7	1.338 (5)	C16—C19	1.504 (8)
N5—C15	1.340 (6)	C17—C18	1.378 (8)
N5—C18	1.344 (6)	C17—H17	0.9300
N6—C15	1.332 (6)	C18—C20	1.493 (7)
N6—C16	1.337 (6)	C19—H19A	0.9600
N7—C21	1.340 (5)	C19—H19B	0.9600
N7—C24	1.341 (6)	C19—H19C	0.9600
N8—C21	1.330 (5)	C20—H20A	0.9600
N8—C22	1.331 (6)	C20—H20B	0.9600
S1—C1	1.752 (5)	C20—H20C	0.9600
S2—C7	1.749 (5)	C22—C23	1.380 (6)
S3—C15	1.744 (5)	C22—C25	1.494 (6)
S4—C21	1.751 (5)	C23—C24	1.372 (7)
C2—C3	1.358 (7)	C23—H23	0.9300
C2—C5	1.508 (7)	C24—C26	1.506 (7)
C3—C4	1.382 (7)	C25—H25A	0.9600
C3—H3	0.9300	C25—H25B	0.9600
C4—C6	1.486 (7)	C25—H25C	0.9600
C5—H5A	0.9600	C26—H26A	0.9600
C5—H5B	0.9600	C26—H26B	0.9600
C5—H5C	0.9600	C26—H26C	0.9600
C6—H6A	0.9600	C27—H27A	0.9600
C6—H6B	0.9600	C27—H27B	0.9600
C6—H6C	0.9600	C27—H27C	0.9600
C8—C9	1.374 (7)	C28—H28A	0.9600
C8—C11	1.504 (7)	C28—H28B	0.9600
C9—C10	1.366 (7)	C28—H28C	0.9600
			
C14—Sn1—C13	125.7 (3)	Sn1—C13—H13A	109.5
C14—Sn1—S1	109.25 (18)	Sn1—C13—H13B	109.5
C13—Sn1—S1	109.44 (18)	H13A—C13—H13B	109.5
C14—Sn1—S2	109.59 (17)	Sn1—C13—H13C	109.5
C13—Sn1—S2	108.57 (16)	H13A—C13—H13C	109.5
S1—Sn1—S2	87.70 (5)	H13B—C13—H13C	109.5
C27—Sn2—C28	125.9 (2)	Sn1—C14—H14A	109.5
C27—Sn2—S4	106.60 (15)	Sn1—C14—H14B	109.5
C28—Sn2—S4	112.26 (16)	H14A—C14—H14B	109.5
C27—Sn2—S3	107.81 (16)	Sn1—C14—H14C	109.5
C28—Sn2—S3	108.97 (16)	H14A—C14—H14C	109.5
S4—Sn2—S3	88.93 (4)	H14B—C14—H14C	109.5
C4—N1—C1	117.6 (4)	N6—C15—N5	126.9 (4)
C1—N2—C2	115.1 (4)	N6—C15—S3	117.5 (4)
C10—N3—C7	117.3 (4)	N5—C15—S3	115.6 (3)
C8—N4—C7	115.6 (4)	N6—C16—C17	121.6 (5)
C15—N5—C18	116.3 (4)	N6—C16—C19	115.2 (6)
C15—N6—C16	115.6 (5)	C17—C16—C19	123.2 (6)
C21—N7—C24	116.4 (4)	C16—C17—C18	119.6 (5)
C21—N8—C22	116.0 (4)	C16—C17—H17	120.2
C1—S1—Sn1	91.65 (16)	C18—C17—H17	120.2
C7—S2—Sn1	93.76 (17)	N5—C18—C17	119.9 (5)
C15—S3—Sn2	93.08 (17)	N5—C18—C20	116.2 (5)
C21—S4—Sn2	92.63 (16)	C17—C18—C20	123.9 (6)
N2—C1—N1	126.9 (4)	C16—C19—H19A	109.5
N2—C1—S1	117.8 (4)	C16—C19—H19B	109.5
N1—C1—S1	115.3 (3)	H19A—C19—H19B	109.5
N2—C2—C3	121.9 (5)	C16—C19—H19C	109.5
N2—C2—C5	115.1 (6)	H19A—C19—H19C	109.5
C3—C2—C5	123.0 (6)	H19B—C19—H19C	109.5
C2—C3—C4	119.8 (5)	C18—C20—H20A	109.5
C2—C3—H3	120.1	C18—C20—H20B	109.5
C4—C3—H3	120.1	H20A—C20—H20B	109.5
N1—C4—C3	118.6 (5)	C18—C20—H20C	109.5
N1—C4—C6	117.4 (5)	H20A—C20—H20C	109.5
C3—C4—C6	123.9 (5)	H20B—C20—H20C	109.5
C2—C5—H5A	109.5	N8—C21—N7	126.9 (4)
C2—C5—H5B	109.5	N8—C21—S4	118.1 (4)
H5A—C5—H5B	109.5	N7—C21—S4	115.0 (3)
C2—C5—H5C	109.5	N8—C22—C23	121.3 (5)
H5A—C5—H5C	109.5	N8—C22—C25	116.6 (5)
H5B—C5—H5C	109.5	C23—C22—C25	122.1 (5)
C4—C6—H6A	109.5	C24—C23—C22	118.9 (5)
C4—C6—H6B	109.5	C24—C23—H23	120.5
H6A—C6—H6B	109.5	C22—C23—H23	120.5
C4—C6—H6C	109.5	N7—C24—C23	120.5 (4)
H6A—C6—H6C	109.5	N7—C24—C26	116.5 (5)
H6B—C6—H6C	109.5	C23—C24—C26	123.0 (5)
N3—C7—N4	126.3 (5)	C22—C25—H25A	109.5
N3—C7—S2	117.0 (4)	C22—C25—H25B	109.5
N4—C7—S2	116.7 (4)	H25A—C25—H25B	109.5
N4—C8—C9	121.3 (4)	C22—C25—H25C	109.5
N4—C8—C11	116.4 (5)	H25A—C25—H25C	109.5
C9—C8—C11	122.3 (5)	H25B—C25—H25C	109.5
C10—C9—C8	119.1 (5)	C24—C26—H26A	109.5
C10—C9—H9	120.4	C24—C26—H26B	109.5
C8—C9—H9	120.4	H26A—C26—H26B	109.5
N3—C10—C9	120.4 (5)	C24—C26—H26C	109.5
N3—C10—C12	117.4 (5)	H26A—C26—H26C	109.5
C9—C10—C12	122.2 (6)	H26B—C26—H26C	109.5
C8—C11—H11A	109.5	Sn2—C27—H27A	109.5
C8—C11—H11B	109.5	Sn2—C27—H27B	109.5
H11A—C11—H11B	109.5	H27A—C27—H27B	109.5
C8—C11—H11C	109.5	Sn2—C27—H27C	109.5
H11A—C11—H11C	109.5	H27A—C27—H27C	109.5
H11B—C11—H11C	109.5	H27B—C27—H27C	109.5
C10—C12—H12A	109.5	Sn2—C28—H28A	109.5
C10—C12—H12B	109.5	Sn2—C28—H28B	109.5
H12A—C12—H12B	109.5	H28A—C28—H28B	109.5
C10—C12—H12C	109.5	Sn2—C28—H28C	109.5
H12A—C12—H12C	109.5	H28A—C28—H28C	109.5
H12B—C12—H12C	109.5	H28B—C28—H28C	109.5

### Hydrogen-bond geometry (Å, °)

**Table:**

*D*—H···*A*	*D*—H	H···*A*	*D*···*A*	*D*—H···*A*
C6—H6A···N6^i^	0.96	2.55	3.397 (7)	147.

Symmetry codes: (i) −*x*+1/2, *y*−1/2, −*z*+1/2.

## References

[bb1] Duboy, S. K. & Roy, U. (2003). *Appl. Organomet. Chem.* **17**, 3–8.

[bb2] Gielen, M. (2002). *Appl. Organomet. Chem.* **16**, 481–494.

[bb3] Sheldrick, G. M. (1996). *SADABS* University of Göttingen, Germany.

[bb4] Sheldrick, G. M. (2008). *Acta Cryst.* A**64**, 112–122. 10.1107/S0108767307043930 18156677

[bb5] Siemens (1996). *SMART* and *SAINT* Siemens Analytical X-ray Instruments Inc., Madison, Wisconsin, USA.

